# Service Needs for Corrections-Involved Parents With a History of Problematic Opioid Use: A Community Needs Assessment

**DOI:** 10.3389/fpsyg.2021.667389

**Published:** 2021-10-21

**Authors:** Miriam Clark, Jean Kjellstrand, Kaycee Morgan

**Affiliations:** UO Criminal Justice Lab, College of Education, Counseling Psychology and Human Services/Prevention Science, University of Oregon, Eugene, OR, United States

**Keywords:** incarceration, reentry, parenting, opioid use, parental incarceration

## Abstract

The incarceration of a parent is often a continuation of a challenging family situation marked by poverty, unstable housing, trauma, and abuse. These challenges make it difficult for incarcerated parents reentering their communities to raise their children effectively and, thus, increase the likelihood of poor outcomes for their children. Children whose parents are also battling opioid misuse have an even higher risk for long-term problems. This study uses survey data from 48 community service providers to better understand the service needs of parents with histories of problematic opioid use who are reentering their communities after incarceration. Community service providers recommended implementing intervention programs that cover critical information related to basic needs, supportive community resources, drug treatment programs, and parenting to help individuals thrive in their communities and meet their children's needs. The services most frequently identified by providers as important for reentering parents included housing, mentors or peer counselors, mental health support, group therapy and other support programs. Key topics to address in parenting programs included problem-solving techniques, the effect of parent's addiction on children, and strategies for connecting with and meeting children's needs. Suggestions are made for future research and intervention development.

## Introduction

Over the past four decades, the United States has led the world in both the rate and number of incarcerated individuals (Walmsley, 2018). More than half of the inmates held in U.S. state or federal prison are parents to minor children (Glaze and Maruschak, 2009). On any given day, ~4% of U.S. minors have an incarcerated parent (Sykes and Pettit, 2014), with millions more experiencing parental incarceration at some time during their childhood (Glaze and Maruschak, 2009; Murphey and Cooper, 2015). This prison boom has not only affected the incarcerated parents, but also their children and families who were left behind to struggle with family disruption, diminished financial resources, and emotional strain. Disproportionately impacted are populations of color and other marginalized populations (Tucker, 2014). Nearly all incarcerated parents will return to their communities (Carson and Anderson, 2016). When they do, many face a host of complex and long-term challenges, including substance abuse, mental illness, poverty, discrimination, unemployment, physical health problems, and eroded family and social networks (Arditti and Few, 2006; Brown and Bloom, 2009; Kjellstrand and Eddy, 2011b).

Reentry into their communities can be particularly challenging for the nearly 20% of corrections-involved parents meeting the criteria for an opioid use disorder (OUD) before incarceration (Joudrey et al., 2019). When incarcerated, these individuals are forced to detox—often without medical intervention—and typically do not receive any substance use treatment during prison (Nunn et al., 2009). Without proper rehabilitation, many struggle with opioid use after reentering their communities. In fact, in the period immediately after release from prison, individuals with histories of problematic opioid use are at high risk of overdosing due to their lower drug tolerance after forced abstinence during incarceration, combined with inaccessibility to treatment (Nunn et al., 2009; Binswanger et al., 2013). When the individuals are parents, the misuse of opioids can lead to poor outcomes for their children (Geller et al., 2009; Kjellstrand and Eddy, 2011a; Murray et al., 2012b; Peisch et al., 2018).

Although research continues to grow about the effects of parental incarceration and parental opioid misuse on children, little is known regarding how to best support corrections-involved parents with a history of problematic opioid use (CIO parents), their children, and their families during incarceration and after release. This study seeks to understand the service needs of CIO parents by soliciting views from community service providers who work with CIO parents during reentry.

## Background

Reentry from prison back into the community can be difficult for those who have been incarcerated (Hughes and Wilson, 2003; Morenoff and Harding, 2014). Many struggle with problems they faced before incarceration, such as low education levels, poverty, discrimination, underemployment, and dysfunctional relationships (Mumola, 2000; Petersilia, 2003; Glaze and Maruschak, 2009). Moreover, some return to social networks that endorse the commission of criminal and harmful behaviors (e.g., Dodge et al., 2007; Boman and Mowen, 2017), are strained and conflictual (e.g., Greene et al., 2000; James and Glaze, 2006; Kjellstrand and Eddy, 2011a; Wallace et al., 2016), or have eroded due to time apart (Rabuy and Kopf, 2015). Because of their criminal records, the reentering adults often face new challenges related to securing employment; finding safe, affordable housing; and dealing with marginalization, biases, and disadvantage (Travis, 2005; Brazzell et al., 2009; Hamilton-Smith and Vogel, 2012). For CIO parents, the challenges are even greater. As parents attempt to reconnect with their children and family members from whom they have been separated during incarceration (Travis, 2005), all will face potential relapse due to difficulties accessing treatment during and after incarceration (National Institute on Drug Abuse, 2018; World Health Organization, 2018), and many will deal with issues related to their past problematic substance use, including damaged familial, intimate partner or peer relationships (Daley et al., 2018).

The effect of parental incarceration and parental substance misuse on child development has been well-documented. Children with parents who have been incarcerated are more likely to experience poverty and unstable housing (Geller et al., 2009), have insecure attachments (Murray and Murray, 2010), exhibit antisocial and delinquent behaviors (Kjellstrand and Eddy, 2011a,b; Murray et al., 2012a), suffer from internalizing problems and psychopathology (Foster, 2012; Kjellstrand et al., 2020), have antisocial peers (Cochran et al., 2018), and, as adolescents, misuse drugs, and participate in criminal activities (Geller et al., 2009; Wildeman, 2009; Kjellstrand and Eddy, 2011a,b; Foster and Hagan, 2013). Children of parents who misuse drugs are more likely to incur injuries, experience poor physical and mental health (Raitasalo and Holmila, 2017), exhibit externalizing problem behaviors, and engage in substance use as adolescents (McGovern et al., 2020). Both parental incarceration and parental opioid misuse have been linked to harsh, inconsistent, and disapproving parenting strategies (Kjellstrand and Eddy, 2011b; Peisch et al., 2018). Opioid use, specifically, can negatively affect parental responsiveness and ability to exercise empathy (Richter and Bammer, 2000; Hogan, 2007; Rizzo et al., 2013). These problematic parenting strategies, on top of contextual issues, can worsen child outcomes. Despite past findings on the effect of parental incarceration and parental opioid misuse, few parents receive adequate treatment or support during incarceration or after they are released (Feder et al., 2018; National Institute on Drug Abuse, 2018).

Research on supportive interventions for corrections-involved adults who struggle with opioid use is becoming more prevalent (e.g., Parmar et al., 2016; Marsden et al., 2017; Friedmann et al., 2018). The majority of intervention research points to the importance of medication-assisted treatments (MAT), such as naltrexone at reentry (Gisev et al., 2015; McDonald et al., 2016; Parmar et al., 2016; Marsden et al., 2017; Friedmann et al., 2018; Waddell et al., 2020). Evidence also supports the benefits of individualized treatment and case management for reentering individuals with substance use disorders (Miller et al., 2016; Kendall et al., 2018). However, results are mixed on exactly which interventions are most beneficial (Bitney et al., 2017; Moore et al., 2020).

Unfortunately, research on interventions to support corrections-involved parents—specifically CIO parents—and their families is minimal. Numerous interventions have been implemented in correctional facilities and the community, including such programs as parenting classes, family visitation, prison nurseries, and alternatives to incarceration (Kjellstrand, 2017). However, the effects of these interventions are still largely unknown (Kjellstrand, 2017; Eddy et al., 2019). To the best of our knowledge, no research has focused specifically on how to support CIO parents and their families. Given the immense variation in and complexity of these families, it is unlikely that a “one-size-fits-all” model will provide sufficient support. More research is warranted to better understand the specific needs of CIO parents as well as the most effective ways to support this high-risk population.

Our community assessment of the service needs of CIO parents is a first step toward (1) addressing some of these critical gaps in our knowledge and (2) laying the foundation to build a supportive intervention strategy for CIO parents and their families. By soliciting information from community service providers who work with CIO parents, we take a community-based participatory research approach. Such an approach not only addresses power dynamics and promotes reciprocal knowledge translation (also referred to as ‘knowledge hybridity’), but also allows underrepresented voices a place in research. All of these can help increase the likelihood of successful intervention implementation (Wallerstein and Duran, 2010) and systemic change generation (Collins et al., 2018). In our community needs assessment, we were interested in three specific issues related to service provision: (1) what community service providers see as the most needed services and programs for reentering CIO parents; (2) what community service providers feel CIO parents need to know upon reentry in order to be successful; and (3) what community service providers feel would be most beneficial for CIO parents to learn and practice in a brief parenting intervention to help support them with parenting and reentry challenges.

## Method

### Participants

#### Sampling and Recruitment

After receiving IRB approval, our research team used three distinct methods to recruit primarily Oregon-based social service providers who are familiar with the service needs of CIO parents (e.g., parole and probation officers, mental health, and medical professionals). First, we used snowball sampling where we contacted community service providers with whom we had prior relationships. They, in turn, were asked to suggest other colleagues familiar with our population of interest who might be interested in participating in our study. Community partners were provided an overview and goals of the project and then invited to complete an online Qualtrics survey (see Appendix A). This approach yielded 26 respondents.

Second, we sent out individual emails and website queries to additional service providers identified using internet search engines, county websites, and online community resource sheets. All emails and website queries included information on the project, a link to the survey, and a request to forward the email to other colleagues who worked with this population. A total of 53 agencies were invited to participate via an agency online website query form, and an additional 196 individuals were invited via email using the contact information found online. We received 29 automated responses informing us that those email addresses were invalid. Twelve individuals responded to the email but declined to participate. Follow-up emails were sent to the remaining 88 individuals ~1 week after the initial email to remind them of the survey. Last, we posted a link to the survey on Twitter and Facebook and invited service providers familiar with the population to participate. In the end, a total of 48 service providers completed the survey.

#### Sample

Table 1 displays demographic information for the respondents.

**Table 1:** Demographic information.

**Variable**	**Mean**	**SD**	**%**
**Age**	47.98	11.57	
**Race/Ethnicity**			
White			85.11
Hispanic, latino, or Spanish Origin			8.51
American indian or alaska native			8.51
Black or African American			4.26
Other			4.26
Asian			2.13
Native Hawaiian or other Pacific Islander			0.00
**Gender**			
Female			63.83
Male			34.04
Decline			2.13
**Education level**			
Some college credit with no degree			4.26
Associates degree			10.64
Bachelor's degree			31.91
Master's degree			38.30
Professional degree			4.26
Doctorate degree			10.64

Respondents worked in a variety of fields and positions including addiction support (e.g., addictions clinical supervisor, alcohol and drug prevention coordinator), health care (e.g., community health worker, psychiatrist), the corrections system (e.g., parole and probation officers, judge), and mental health (e.g., clinical social worker, clinical director). Additionally, one researcher, one licensed minister, and one author completed the survey. The majority of participants had worked in their respective fields for over 15 years.

#### Survey Instrument

An online Qualtrics survey was used to gather input from the respondents. The survey contained five main sections. In the first section, participants were asked to provide basic demographic information (e.g., education level, race, ethnicity, occupation). Participants were then asked open-ended questions regarding what they viewed as needs or gaps in services for CIO parents. Following this section, participants were invited to review a set of potential topics and activities for a parenting intervention program and indicate which they felt would be beneficial for CIO parents. These topics were derived from a previously developed intervention for a similar population (see Eddy et al., 2019). In this section of the survey, they also indicated what they felt were the three most important parenting topics to address as well as the three most important activities to include in a brief intervention. Participants were able to suggest topics and activities that were not listed on the survey. Finally, participants were given the option to elaborate on any of the topics or activities they desired in an open-ended short answer format. Responses to all questions were optional; most participants chose to skip at least some of the questions. On average, participants took 10–30 min to complete the brief survey.

### Analysis

All data were exported from Qualtrics to IBM SPSS Statistics 26. Descriptive analyses were run on all the quantitative questions. The open-ended short answer questions were coded manually—grouping similar themes into categories and calculating the respective frequencies. Responses were collaboratively discussed among research team members to ensure the reliability of the codes (see Sweeny et al., 2012 for information on this consensus-building approach).

## Results

### Needed Services and Programs

Participants identified eighteen different supports that they felt CIO individuals needed to successfully reenter the community. The services and supports tended to address three specific needs: *basic reentry needs, needs related to problematic opioid use*, and *parenting specific needs* (see Table 2). Some of the most frequently identified services included: housing, mentors or peer counselors, mental health support, treatment services, additional MAT facilities, parenting education or programs, advocates to help navigate Department of Health and Human Services (DHS)/ child custody/ reunification, childcare, and resources for the child.

**Table 2:** Needed supports for CIO parents.

**Type of support**	**Percent of participants that listed each type of support**
**Supports to address basic re-entry needs**	
Housing	39.58
Mentors or peer counselors	12.50
Mental health support	10.41
Group therapy or support programs	8.33
Employment resources	8.33
Additional case management	8.33
Additional resources during incarceration	6.25
Social support	6.25
Additional collaboration between programs	6.25
Transportation	4.17
Cell phone for reentering individuals	2.08
Early intervention for children with CIO parents	2.08
Additional research to better understand issues faced by CIO parents	2.08
Trauma-informed care for CIO parents	2.08
Adult Education	2.08
**Supports related to problematic opioid use**	
Treatment services	18.75
Additional MAT facilities	6.25
**Supports to address parenting specific needs**	
Parenting education/programs	10.41
Advocates to help navigate DHS/ child custody/reunification	6.25
Childcare	4.17
Resources for the child	2.08

*CIO, corrections-involved with a history of problematic opioid use; MAT, medication-assisted treatments; DHS, Department of Health and Human Services*.

### Important Knowledge for CIO Parents

When asked what CIO parents needed to know upon reentry, community service providers identified eighteen unique topics, which—similar to the previous section—tended to fall in three specific areas: basic reentry needs (e.g., information on positive social networks, housing, employment, and general resources); problematic opioid use (e.g., information on treatment programs, recovering from addiction, and developing a recovery plan); and parenting (e.g., information on parenting, childcare, and family counseling). See Table 3 for a complete list of the items mentioned.

**Table 3:** Needed knowledge for CIO parents.

**Type of Knowledge**	**Percent of participants that listed each type of knowledge**
**Knowledge to address basic re-entry needs**	
Positive social networks	35.42
Information or case management for general resource connection	25.00
Housing resources	25.00
Job resources	14.58
Mental health services or individual therapy resources	14.58
Peer support resources	12.50
Relationship skills training and resources	6.25
Positive relationships with probation officer	6.25
Skills and strategies for self-care	4.17
Transportation resources	2.08
Physical health resources	2.08
**Knowledge related to problematic opioid use**	
Treatment program resources	27.08
MAT resources	20.83
Understanding of the time and work necessary to recover from addiction	10.42
Developing a recovery plan	6.25
**Knowledge to address parenting specific needs**	
Information about parenting resources	12.50
Information about childcare resources	6.25
Family counseling resources	2.08

*CIO, corrections-involved with a history of problematic opioid use; MAT, medication-assisted treatments*.

### Parenting Intervention Knowledge and Activities

The next section of the survey examined topics and activities that service providers felt would be important to present in a parenting intervention (see Table 4). Participants were provided a list of twelve potential topics and eight potential activities and asked to mark any items on the list they felt would be important to include in a parenting intervention. They were also given the opportunity to identify other parenting topic and activities they deemed important. Out of the topics and activities that they had marked as important, they were then asked to identify the three most important topics and the three most important activities. We utilized a technique in Qualtrics so that participants could only select the top three most important topics and activities from those that they had already marked as important. Topics that were most likely to be identified by service providers as one of the three most important included: problem-solving techniques (*n* = 26), the effect of parental addiction's on children (*n* = 22), specific strategies for connecting with and meeting children's needs (*n* = 20), appropriate self-care management techniques (*n* = 19), and strategies for managing the effects of trauma (*n* = 19). Activities listed by service providers as one of the three most important for CIO parents included: working with a parent coach to develop a plan for solving problems (*n* = 35), working with a parent coach to implement specific strategies for connecting with and meeting children's needs (*n* = 31), and role-playing difficult conversations with child, partner, or others (*n* = 27).

**Table 4:** Important topics and activities for reentering parents with opioid addiction.

**Learning topic for parenting**	**Percent of participants indicating topic is important to address**	**Percent of participants ranking topic as one of three most important to address**
Problem-solving techniques.	89.58	54.17
Parental addiction's effect on children.	89.58	45.83
Strategies to connect with or meet children's needs.	87.50	41.67
Managing effects of trauma.	87.50	39.58
Appropriate self-care management techniques.	85.42	39.58
Family strengths and challenges during reentry.	85.42	25.00
Basics of mindfulness (e.g., deep breathing, fully present, meditation).	81.25	31.25
Appropriate routines to engage in with children.	77.08	16.67
Age appropriate activities to engage with children.	70.83	10.42
Issues related to domestic violence.	70.83	6.25
Building a relationship with the child's caregiver.	60.42	12.50
Personal parental hopes.	60.42	2.08
**Hands-on Activity for Parenting**	**Percent of Participants Indicating Activity is Important to Do**	**Percent of Participants Ranking Activity as One of Three Most Important to Do**
Practice strategies to connect with or meet children's needs.	91.67	64.58
Develop plan to solve problems.	87.50	72.92
Role-play difficult conversations with child, partner, or others.	77.08	56.25
Role-play difficult situations around opioid use.	68.75	41.67
Work on age-appropriate routines to engage in with child.	62.50	22.92
Practice age-appropriate activities to engage with children.	60.42	8.33
Engage in mindfulness meditation.	47.92	14.58
Engage in mindfulness meditation with child.	41.66	2.08
**Additional topics or activities suggested by participants for intervention**	**Percent of participants suggesting this item**	
*Topics or activities to address basic re-entry needs*		
Learn how to access resources	20.83	
Self-care and practice	12.50	
Connect with peers/peer support	12.50	
Availability and access to mental health support	12.50	
Learn criminogenic risk/need	8.33	
Learn communication skills	6.25	
Learn self-advocacy	4.17	
Learn about trauma	4.17	
Learn financial management or job search skills	4.17	
*Topics or activities related to problematic opioid use*		
Addiction recovery knowledge or support	14.58	
Engagement in a 12-step program	4.17	
In prison addiction treatment	2.08	
*Topics or activities to address parenting specific needs*		
Learn child needs	10.42	
Learn child advocacy	4.17	

Finally, we asked participants in an open-ended format to list other activities they felt were important for CIO parents who are exiting the prison system to either learn or do in an intervention program. Participants identified seventeen different topics and activities. The most frequently identified items related to basic reentry needs included gaining access to resources, learning and practicing self-care strategies, connecting with peers or peer support, and accessing mental health support. Items connected to problematic opioid use included learning about and practicing strategies related to addiction recovery, accessing addiction or other types of support, engaging in a 12-step program, and accessing in-prison substance use treatment. Finally, two items related to parenting that were not mentioned in the earlier list of potential topics included learning more about child needs and learning how to advocate for their child. In these short, open-ended responses, most participants did not provide extensive details on what they envisioned for these topics or activities.

## Discussion

Reentry can be a difficult time, fraught with economic difficulties (Mumola, 2000; Petersilia, 2003; Glaze and Maruschak, 2009), strained social networks (Greene et al., 2000; James and Glaze, 2006; Wallace et al., 2016), and societal stigma and disadvantages (Alexander, 2020). Some of the most reported challenges facing incarcerated parents concern securing employment, finding quality affordable housing, maintaining good physical and mental health, and developing healthy relationships (Gaes and Kendig, 2003; Kjellstrand, 2017). CIO parents face additional difficulties as they manage issues related to problematic substance use (Winkelman et al., 2018; Gannon et al., 2020) and navigate strained relationships with their children and families (Mirick and Steenrod, 2016; Stulac et al., 2019).

The purpose of our study was to gain a better understanding of the reentry needs of CIO parents from the perspectives of community service providers who work with this population and/or are familiar with the population's needs. Such perspectives are essential in guiding the development of effective and relevant interventions for these parents and families. To our knowledge, this is the only study that has examined the needs of reentering CIO parents from the perspective of community service providers.

Our findings underscore the multiple challenges CIO parents encounter in three central areas related to (1) reentry, (2) problematic opioid use, and (3) parenting. Further, our findings point to some of the topics and activities in each of these areas that community service providers feel would be most beneficial for CIO parents as they return to their families and communities after incarceration.

Community service providers in our sample showed a deep understanding of the issues that CIO parents faced during reentry. They described a variety of basic reentry needs and stressed the importance of CIO parents knowing where to turn to obtain critical information and support in the areas of housing, transportation, physical and mental health, and prosocial relationships. Such information and support can significantly improve outcomes for individuals post-incarceration in multiple areas and can help promote successful reentry (Visher, 2006; Bahr et al., 2010; Morenoff and Harding, 2014). Without the knowledge of and access to such resources, reentering individuals may struggle, relapse, or recidivate.

In terms of problematic opioid use, community service providers suggested many evidence-based supports and treatments. For example, several of our participants listed the importance of MAT for CIO parents, echoing research demonstrating the value of such treatment for reentering individuals in preventing use and potential overdose (Gisev et al., 2015; McDonald et al., 2016; Marsden et al., 2017; Friedmann et al., 2018; Waddell et al., 2020). Additionally, community service providers stressed the importance of specific individualized treatment, consistent with findings that such treatment can be beneficial for reentering individuals struggling with problematic opioid use (Miller et al., 2016). However, community service providers in our sample had vast opinions on what specific treatment would be most helpful for CIO parents (e.g., group therapy or support programs, mentors or peer counselors, trauma-informed care). Given that research is mixed on which types of individualized treatment are most beneficial (Bitney et al., 2017; Moore et al., 2020), more research is warranted in this area.

Lastly, the community service providers discussed parenting needs and endorsed or identified potential topics and activities they felt would be beneficial to CIO parents for parenting and reentry. Some of the most common topics that participants indicated as important to address in a parenting program included problem-solving techniques, the effects of a parent's addiction on children, strategies for connecting with and meeting children's needs, ways to manage the effects of trauma, and appropriate self-care strategies and management. Activities to support this learning and promote improved parenting and family dynamics included developing a family plan to address problematic issues, practicing strategies to connect with and meet children's needs, role-playing difficult family conversations, developing parenting routines, and practicing mindfulness meditation. Many similar topics and activities arose in a recent study by Kjellstrand (2017) in which incarcerated parents were asked what they needed most to support them in parenting their children both during incarceration and after they returned to their communities. The combined findings highlight the importance and relevance of the identified topics regardless if the parents are struggling with problematic opioid use. Notably, participants tended to mention the need of providing support and knowledge on parenting for CIO parents less frequently than support and knowledge focused on basic reentry needs (e.g., housing, case management, employment, general resources) and opioid use (e.g., treatment programs, MAT, support groups). While parenting is important, based on our results, it might be best to address the topic of parenting as part of a multi-modal program which provides support around critical basic and medical needs of CIO parents or, alternatively, after reentering parents have first attended to their basic needs and secured medical treatment and programming for their opioid use.

## Limitations

Our findings were in line with much of the existing research on supports for individuals who are reentering their communities from prison, providing us with more confidence regarding our results. Although our study provides additional insight into the service needs of CIO parents from the perspective of community service providers, a few limitations must be considered. First, our sample of community service providers was recruited from a specific region of the U.S. Hence, the results may not be generalizable to other geographic regions. Second, our data was collected during the spring and summer of 2020—a time when the world was entering a global pandemic. Many individuals, including community service providers, were navigating difficulties both at work (e.g., shutdowns, agency protocol changes) and at home (e.g., childcare, illness of family members, financial strain). This situation may have biased our sample toward those who were easier to contact or who were experiencing fewer time constraints due to the pandemic. Third, our research does not examine outcome differences by type of provider who might be inclined to stress certain needs over others. Because there were seven different types of providers within our sample of 48, we did not have sufficient numbers of each type to examine statistical differences. A more robust sample could show patterns by type of provider. Fourth, our sample gathered perspectives from a particular group of key stakeholders (i.e., community service providers). Perspectives from other key stakeholders (e.g., individuals and families with lived experience, additional professionals in the corrections system) could provide further insight into the needs of CIO parents as they return to their communities after prison. Last, because our study was meant to inform an intervention for a particular population, the survey was made specifically for this study and did not use validated measures.

## Implications

Despite these limitations, our findings provide valuable insight for the development of a supportive strategy to meet the needs of CIO parents and their families. The community service providers highlighted the importance of addressing parenting but with an eye toward each individual's reentry needs and context. Successful reentry will look different depending on the circumstances of the parent and family. Ideally, a reentry program would begin supporting parents while they are still incarcerated, providing key information, treatment, and transitional planning to ensure that each parent and their family have relevant tools and knowledge as well as a strong comprehensive support system in place before the parent leaves prison. Such support would then continue as the parents returned and reintegrated into their communities and families. Reentry is a long process, and, given limited community resources, it can be challenging for a single program or organization to address the complex needs faced by CIO parents during reentry. Creating a multi-modal strategy that links relevant programs might be the most economical and efficient way to provide thorough support for individuals. Key programs to include in such a system would provide support around three critical areas identified in our study: general reentry needs, substance use, and parenting. Establishing such organized systems of care could streamline the process for reentering CIO parents and enable these individuals to more easily access the knowledge and support they need. In respect to parenting specifically, a program that addresses basic parenting strategies, the effects of parental incarceration and opioid use on child development, and self-care management techniques could be especially valuable and relevant for CIO parents. Providing supplemental activities that afford parents the opportunity to develop plans and practice specific skills could help improve parents' understanding, retention of the content, and likelihood of implementing the parenting strategies.

## Conclusion

Reentry from prison to the community can be challenging for everyone, but particularly for CIO parents. In our study, community service providers highlighted the importance of providing CIO parents with the knowledge and skills needed to navigate this difficult period especially in terms of addressing their basic needs, handling problematic substance use, and parenting their children effectively. Given the complex needs facing CIO parents and their families, this might be done most effectively through a collaborative approach across systems and at different points during incarceration and reentry. We believe that such a strategy will lead to better outcomes not only for reentering parents, but for their children and families as well.

## Data Availability Statement

The raw data supporting the conclusions of this article will be made available by the authors, without undue reservation.

## Ethics Statement

The studies involving human participants were reviewed and approved by the University of Oregon's Institutional Review Board. The patients/participants provided their written informed consent to participate in this study.

## Author Contributions

MC and JK conceived, conceptualized, and designed the study. JK acquired funding and resources. MC gathered and analyzed the data. MC, JK, and KM drafted the manuscript. All authors contributed to the article and approved the submitted version.

## Funding

The research was supported by funds from the University of Oregon: Counseling Psychology and Human Services Department. Publication of this work was supported by the University of Oregon.

## Conflict of Interest

The authors declare that the research was conducted in the absence of any commercial or financial relationships that could be construed as a potential conflict of interest.

## Publisher's Note

All claims expressed in this article are solely those of the authors and do not necessarily represent those of their affiliated organizations, or those of the publisher, the editors and the reviewers. Any product that may be evaluated in this article, or claim that may be made by its manufacturer, is not guaranteed or endorsed by the publisher.
