# Supplementary material for: Service Needs for Corrections-Involved Parents With a History of Problematic Opioid Use: A Community Needs Assessment
Source: Front Psychol. 2021 Oct 21;12:667389. doi: 10.3389/fpsyg.2021.667389 (PMC8566547; doi:10.3389/fpsyg.2021.667389)
Supplement: Supplementary file 1 [file Data_Sheet_1.PDF]

Appendix A: QUESTIONNAIRE

- DEMOGRAPHICS SECTION
  1. Gender
     - ☐ Male
     - ☐ Female
     - ☐ Non-Binary
     - ☐ Transgender
     - ☐ Other (Please describe)
  2. Age
     - ☐ In years _________________
  3. Ethnicity: Are you of Hispanic, Latino, or Spanish origin?
     - ☐ Yes
     - ☐ No
  4. Race: How would you describe yourself? (check all that apply)
     - ☐ White
     - ☐ Black or African-American
     - ☐ American Indian or Alaskan Native
     - ☐ Asian
     - ☐ Native Hawaiian or other Pacific Islander
     - ☐ Other (please specify)
  5. Education level
     - ☐ No schooling completed
     - ☐ 6th grade or less
     - ☐ 7th or 8th grade
     - ☐ Some high school, no HS diploma or GED
     - ☐ High school graduate
     - ☐ GED or equivalent
     - ☐ Some college credit, no degree
     - ☐ Trade/technical/vocational training
     - ☐ Associate degree
     - ☐ Bachelor's degree
     - ☐ Master's degree
     - ☐ Professional degree
     - ☐ Doctorate degree

6. Current job title__________________________

7. How long have you been working in your current position?
   - ☐ Less than one year
   - ☐ 1-2 years
   - ☐ 3-4 years
   - ☐ 5-6 years
   - ☐ 7-10 years
   - ☐ 11-15 years
   - ☐ 16-20 years
   - ☐ 21-25 years
   - ☐ More than 25 years

8. How long have you been working in your field?
   - ☐ Less than one year
   - ☐ 1-2 years
   - ☐ 3-4 years
   - ☐ 5-6 years
   - ☐ 7-10 years
   - ☐ 11-15 years
   - ☐ 16-20 years
   - ☐ 21-25 years
   - ☐ More than 25 years

9. In which city, county, and state do you work?

10. What is the name of your employer? ________________________

11. What do corrections-involved parents with histories of an opioid use disorder or opioid misuse need or need to know for them to be successful as they reenter the community after incarceration? (briefly describe)

12. Please list services or programs that are currently available in your community which are helpful to this population

13. Please list services or programs that are needed in your community.

- QUESTIONS ABOUT OPINIONS ON MODULE TOPICS

1. Which of the following **topics** do you think would be important to cover in an intervention for parents with histories of an opioid use disorder or opioid misuse who are exiting the prison system? [Check all that apply]
   - □ LEARNING: Gain awareness of family strengths and challenges during reentry.
   - □ LEARNING: Gain insight into personal parental hopes.
   - □ LEARNING: Discuss specific strategies to connect with and meet children's needs.
   - □ LEARNING: Learn age-appropriate activities to engage with children.
   - □ LEARNING: Gain understanding of appropriate routines to engage in with children.
   - □ LEARNING: Build relationship with the child's caregiver
   - □ LEARNING: Problem solving techniques
   - □ LEARNING: Manage impacts of trauma
   - □ LEARNING: Gain understanding of parental addiction's impact on children
   - □ LEARNING: Learn appropriate self-care management techniques.
   - □ LEARNING: Learn basics of mindfulness (e.g., deep breathing, fully present, meditation)
   - □ LEARNING: Gain understanding of issues related to domestic violence.
   - □ Other ___________________

2. Of these **topics**, which are the three most important for this population [Check three]
   - □ LEARNING: Gain awareness of family strengths and challenges during reentry.
   - □ LEARNING: Gain insight into personal parental hopes.
   - □ LEARNING: Discuss specific strategies to connect with and meet children's needs.
   - □ LEARNING: Learn age-appropriate activities to engage with children.
   - □ LEARNING: Gain understanding of appropriate routines to engage in with children.
   - □ LEARNING: Build relationship with the child's caregiver
   - □ LEARNING: Problem solving techniques
   - □ LEARNING: Manage impacts of trauma
   - □ LEARNING: Gain understanding of parental addiction's impact on children

- ☐ LEARNING: Learn appropriate self-care management techniques.
- ☐ LEARNING: Learn basics of mindfulness (e.g., deep breathing, fully present, meditation)
- ☐ LEARNING: Gain understanding of issues related to domestic violence.
- ☐ Other ___________________

3. Which of the following **activities** do you think would be important to do during an intervention for parents with histories of an opioid use disorder or opioid misuse who are exiting the prison system?
   - ☐ HANDS-ON: Work with a parent coach to implement specific strategies to connect with and meet children's needs.
   - ☐ HANDS-ON: Work with a parent coach to implement age-appropriate activities to engage with children.
   - ☐ HANDS-ON: Work with a parent coach to implement appropriate routines to engage in with child.
   - ☐ HANDS-ON: Engage in mindfulness meditation.
   - ☐ HANDS-ON: Engage in mindfulness meditation with the child.
   - ☐ HANDS-ON: Role play difficult conversations with child, partner, or others
   - ☐ HANDS-ON: Role play difficult situations around opioid use
   - ☐ HANDS-ON: Work with parent coach to develop plan to solve problems
   - ☐ Other ___________________

4. What other **activities** would be important to do in an intervention for parents with histories of an opioid use disorder or opioid misuse who are exiting the prison system? ___________________________

5. Of these **activities**, which are the three most important for this population [Check three]
   - ☐ HANDS-ON: Work with a parent coach to implement specific strategies to connect with and meet children's needs.
   - ☐ HANDS-ON: Work with a parent coach to implement age appropriate activities to engage with children.
   - ☐ HANDS-ON: Work with a parent coach to implement appropriate routines to engage in with child.
   - ☐ HANDS-ON: Engage in mindfulness meditation.
   - ☐ HANDS-ON: Engage in mindfulness meditation with the child.
   - ☐ HANDS-ON: Role play difficult conversations with child, partner, or others
   - ☐ HANDS-ON: Role play difficult situations around opioid use

- ☐ HANDS-ON: Work with parent coach to develop plan to solve problems
- ☐ Other ___________________

- SHORT ANSWER QUESTIONS
  1. Is there anything specific about the following topics or activities that you feel should be especially emphasized? Feel free to answer whichever ones you'd like to answer and leave blank any as well.
     - ☐ LEARNING: Gain awareness of family strengths and challenges during reentry.

       ____

     - ☐ LEARNING: Gain insight into personal parental hopes.

       ____

     - ☐ LEARNING: Discuss specific strategies to connect with and meet children's needs.

       ____

     - ☐ LEARNING: Learn age-appropriate activities to engage with children.

       ____

     - ☐ LEARNING: Gain understanding of appropriate routines to engage in with children.

       ____

     - ☐ LEARNING: Gain understanding of parental addiction's impact on children.

       ____

     - ☐ LEARNING: Learn appropriate self-care management techniques.

       ____

     - ☐ LEARNING: Learn basics of mindfulness (e.g., deep breathing, fully present, meditation).

       ____

     - ☐ HANDS-ON: Work with a parent coach to implement specific strategies to connect with and meet children's needs.

       ____

     - ☐ HANDS-ON: Work with a parent coach to implement age-appropriate activities to engage with children.

       ____

     - ☐ HANDS-ON: Work with a parent coach to implement appropriate routines to engage in with child.

       ____

     - ☐ HANDS-ON: Engage in mindfulness meditation.

       ____

- ☐ HANDS-ON: Engage in mindfulness meditation with the child.

  ____

Thank you for taking the time to respond to this survey!
